# The Impact of Intraoperative CT-Based Navigation in Congenital Craniovertebral Junction Anomalies: New Concepts of Treatment

**DOI:** 10.3390/brainsci14121228

**Published:** 2024-12-06

**Authors:** Giorgio Cracchiolo, Ali Baram, Gabriele Capo, Zefferino Rossini, Marco Riva, Andrea Fanti, Mario De Robertis, Maurizio Fornari, Federico Pessina, Carlo Brembilla

**Affiliations:** 1 School of Medicine and Surgery, University of Milano-Bicocca, 24127 Bergamo, Bergamo, Italy; g.cracchiolo@campus.unimib.it; 2 Department of Neurosurgery, ASST Papa Giovanni XXIII, 24127 Bergamo, Bergamo, Italy; fanti.andrea@gmail.com; 3 Department of Neurosurgery, IRCSS Humanitas Research Hospital, Via Alessandro Manzoni 56, 20089 Rozzano, Milan, Italy; ali.baram@humanitas.it (A.B.); gabriele.capo@humanitas.it (G.C.); zefferino.rossini@humanitas.it (Z.R.); marco.riva@hunimed.eu (M.R.); mario.derobertis@humanitas.it (M.D.R.); maurizio.fornari@humanitas.it (M.F.); federico.pessina@hunimed.eu (F.P.); 4 Department of Biomedical Sciences, Humanitas University, Via Rita Levi Montalcini 4, 20090 Pieve Emanuele, Milan, Italy

**Keywords:** craniovertebral junction anomalies, navigation, intraoperative imaging, surgical safety, surgical accuracy, posterior fixation

## Abstract

Background: Congenital craniovertebral junction anomalies (CCVJAs) encompass a diverse range of conditions characterized by distorted anatomy and significant variation in the pathways of neurovascular structures. This study aims to assess the safety and feasibility of tailoring posterior fixation for CCVJAs through intraoperative CT-based navigation. Methods: An in-depth retrospective analysis was conducted on eight patients diagnosed with CCVJAs (excluding Arnold–Chiari malformation). These patients underwent posterior fixation/arthrodesis facilitated by intraoperative CT-based navigation. The analysis included an examination of the fixation strategies, complication rates, length of stay, post-operative complications, and success of arthrodesis. Additionally, a comprehensive literature review was undertaken to contextualize and compare our findings. Results: Patients undergoing CVJ posterior fixation with intraoperative CT-based navigation exhibited a flawless record, devoid of complications related to the damage to neurovascular structures, as well as any instances of screw misposition, pullout, or breakage (0 out of 36 total screws). Furthermore, the entire cohort demonstrated a 100% arthrodesis rate. None of the patients required treatment with an occipital plate. Conclusions: The incorporation of intraoperative CT-based navigation proves to be an invaluable asset in executing CVJ posterior fixation within the context of CCVJAs. This technology facilitates the customization of posterior constructs, a crucial adaptation required to navigate the anatomical challenges posed by these anomalies. The secure placement of screws into the occipital condyles, made possible by navigation, has proven highly effective in achieving CVJ fixation, obviating the need for an occipital plate. This technological leap represents a significant advancement, enhancing the safety, precision, and overall outcomes for patients undergoing this surgical procedure, while concurrently reducing the necessity for more invasive and morbid interventions.

## 1. Introduction

The craniovertebral junction (CVJ) serves as a critical anatomical nexus connecting the skull and the cervical spine. This biomechanically complex area includes the occipital condyles (C0), the atlas (C1), the axis (C2), and a network of ligaments crucial for stability, making it the most dynamic part of the cervical spine [1,2].

Since the landmark publication by Goel and Laheri in 1994, which introduced contemporary surgical approaches to this region, the management of CVJ pathologies has evolved significantly [3]. Congenital craniovertebral junction anomalies (CCVJAs) encompass a diverse spectrum of diseases, often characterized by the coexistence of multiple pathologies, presenting significant challenges due to their intricate anatomy [4,5]. These anomalies alter normal anatomical landmarks, potentially leading to confusion during surgery and making the fixation of the region complex and risky for patients [6].

In recent years, technological advancements have transformed the landscape of CCVJA treatment. One particularly important development has been the integration of neuronavigation systems with intraoperative CT scanning. The combination of these technologies has significantly improved the precision and reliability of surgical navigation, allowing for the creation of customized fixation constructs tailored to each patient’s unique anatomy. This approach enhances safety while minimizing the need for more invasive procedures [7,8].

To evaluate the safety and feasibility of using intraoperative CT-based navigation for posterior fixation in CCVJAs, we conducted a retrospective analysis of consecutive patients over a 5-year period at a single institution. In addition, we performed a literature review to compare and contextualize our findings.

## 2. Materials and Methods

### 2.1. Patient Information

For this study, we selected a cohort of 8 complex congenital craniovertebral junction anomaly (CCVJA) cases treated between 2018 and 2023. The cohort included 7 male patients and 1 female patient, with a mean age of 54 years (range: 4 to 78 years). Diagnoses for the 8 cases are detailed in Table 1, based on Menezes classification [9]. The anomalies in this cohort primarily impacted bone tissue. Surgical intervention was performed due to spinal cord or brainstem compression, CVJ instability, and symptoms such as neck pain, as well as neurological deficits including motor weakness, sensory disturbances, and gait or coordination abnormalities.

Arnold–Chiari syndrome was excluded from this series because it is classified as a soft tissue anomaly and often does not necessitate stabilization treatment [10].

### 2.2. Pre-Operative Planning

CT scans and MRI were pivotal in understanding the nature of CCVJAs by delineating anomalous anatomy, identifying potential dislocation of bony structures, and assessing static spinal cord compression. CT scans allowed for the evaluation of anatomical and physical constraints that could impede conventional screw placement, enabling strategic planning for alternative fixation techniques such as C2 laminar screws. Additionally, CT scans provided valuable information about bone quality [11].

Occipital plates were not considered during the planning phase. In cases where occipitocervical stabilization was required, the strategy involved placing screws in C0 or the hybrid C0/C1 condyle, particularly in cases of atlas assimilation.

Dynamic imaging techniques, including MRI and X-ray, played a crucial role in instability assessment, deformity reducibility, and dynamic compression during neck flexion and extension [12]. CT angiography (CTA) or MR angiography (MRA) were essential for mapping the course of the vertebral arteries (VAs), particularly in patients with CCVJAs, where an aberrant VA course is common [6].

### 2.3. Surgical Procedure

Patients were placed in the prone position, and their heads were securely placed in the Mayfield head clamp following gentle traction and reduction maneuvers. The intraoperative CT scan (Medtronic O-arm™ Navigation System, Littleton, MA, USA) and the reference frame for navigation were then prepared.

The incision and exposure of the bony elements were performed according to the standard procedure. Huang et al. [13] highlighted the increased risk of post-operative numbness associated with sacrificing the C2 nerve root, while preserving it may lead to neuralgia. In our practice, ligation and sacrifice of the C2 nerve roots represent the standard of care.

The entry point for the C1 lateral mass screw was determined based on the preservation of normal anatomical landmarks and the extent of anatomical distortion. Bicortical screws were preferred to ensure strong anchorage, while the screw length was carefully chosen to avoid inadvertent damage to the internal carotid artery [14]. In cases of atlas assimilation, where the fused C0–C1 vertebrae exhibited hybrid characteristics, the screw trajectory was carefully monitored to ensure clearance of the aberrant course of the VA and/or the hypoglossal nerve. A typical C1 lateral mass screw had a diameter of 3.5 to 4.0 mm and a length of approximately 36 mm [15].

C2 screws offered versatile placement options, including the pars, pedicles, or laminae [16]. In cases with minimal anatomical distortion, C2 pedicle screws were preferred, typically with a diameter of 3.5 mm and lengths ranging from 15 mm to 30 mm [16]. However, when conditions were unfavorable for C2 pedicle screw fixation, alternative techniques were used, with screws positioned in the pars or laminae. For translaminar screw fixation, a crossed trajectory through the laminae of C2 was employed [17,18]. When C2 fixation was not possible or additional stability was required, C3 pedicle or lateral mass screw fixation was performed.

Arthrodesis presented a significant challenge, due to the limited available bone surfaces. Potential bone graft harvesting sites included the posterior iliac crests and the external diploe of the calvarium [19]. To secure the bone graft onto the posterior elements, classic wiring techniques such as Gallie or Brooks–Jenkins were used, which could also be applied to the occiput by drilling holes into the occipital squama [20,21,22]. Modern solutions like the Jazz™ Lock system, using sublaminar bands, were also utilized. Another technique involved intra-articular arthrodesis between the facet joints of C1 or C0/C1 and C2, which required decorticating the facet joints and filling them with autologous bone chips and/or synthetic bone substitute. In cases where intra-articular gap widening occurred during reduction, the atlantoaxial joint jamming technique was applied, involving the placement of bilateral interbody fusion cages and bone graft material in the intra-articular spaces to ensure biomechanical stability for fusion [23,24].

### 2.4. Complication Avoidance and Follow-Up

Thorough evaluation of pre-operative imaging was crucial for assessing the precise location and abnormal paths of the VAs. The VA is particularly vulnerable to injury during soft tissue dissection around the C1 posterior arch, the placement of C0/C1 screws in cases of atlas assimilation, and the insertion of C2 pedicle screws. Although the hypoglossal canal is typically described as a supracondylar structure, its trajectory may be altered in the context of CCVJAs, increasing the risk of injury during screw insertion [6,25,26].

In cases where pre-operative evaluation revealed significant spinal cord compression or compromise of neural structures, the surgical procedure was performed with intraoperative neurophysiological monitoring, as seen in patients 2 and 7.

Following surgery, patients were immobilized with a rigid cervical collar starting from the first post-operative day for approximately one month, followed by a rehabilitation phase during the subsequent month. Clinical follow-up monitored medium- and long-term complications. Imaging follow-up involved cervical spine CT scans at 1, 3, 6, and 12 months, and then annually. Fusion was evaluated based on the criteria outlined by Tan et al. [27], focusing on documenting bridging osseous union between the proximal and distal endpoints. All patients underwent both imaging and clinical follow-up for a minimum of 12 months.

### 2.5. Search Strategy and Eligibility

A systematic search was conducted across SCOPUS, Medline (PubMed), Cochrane Library, and Google Scholar databases to identify all studies providing relevant information on the use of intraoperative CT scans coupled with navigation in the posterior treatment of CCVJAs. There were no date limits or language restrictions applied during the search. Various combinations of key terms such as “craniovertebral junction anomalies”, “posterior fixation”, and “intraoperative neuronavigation” were used. Additionally, a thorough examination of the references in all included articles was conducted to identify any additional eligible studies. The review included all peer-reviewed case series that reported on the use of intraoperative navigation for the posterior fixation of CCVJAs. Exclusion criteria were as follows: (1) letters to editors and conference abstracts, (2) studies reporting on the use of intraoperative navigation for anterior approaches to the CVJ, (3) studies on the use of intraoperative navigation for posterior fixation of non-malformed vertebrae, and (4) studies on posterior fixation of CCVJAs without the use of intraoperative neuronavigation.

## 3. Results

### 3.1. Surgical Case Series

Intraoperative CT scans with neuronavigation were successfully used for the posterior fixation of eight cases of CCVJAs. All patients underwent rod and screw fixation, with specific techniques tailored to each case. C0/C1 fixation was performed in three cases, C1 lateral mass fixation in five cases, C2 pedicle screw fixation in six cases, and C2 translaminar screw fixation in one case (patient 1). Additionally, C3 pedicle screw fixation was performed in two cases (patients 6 and 8), C3 lateral mass fixation in one case (patient 2), and bilateral 5 mm interbody cages were placed in the C1–C2 articular spaces in one case (patient 1). The Jazz™ Lock system was used in one case (patient 7). Autografts were used in two cases (patients 4 and 7). In all eight cases, correct screw placement and malformation reduction were achieved without injury to the vascular or neural structures. There were no intraoperative or post-operative screw replacements (0 out of 36 screws) due to mispositioning, pullout, or breakage. The average intraoperative surgical time was 209 min. In the immediate post-operative period, one case (patient 6) experienced a dorsal cervicothoracic hematoma on the 14th post-operative day. The average length of stay following surgery was 12 days. The average intraoperative time and length of stay were influenced by patient 8, a polytraumatized patient who underwent multiple surgical interventions in the same session. During the follow-up period, one patient (patient 1) experienced a mild reduction loss without clinical consequences. Arthrodesis was achieved in seven out of eight cases, and osteosynthesis in one case (patient 8), with all eight cases classified as grade I fusions according to Tan et al. [27].

### 3.2. Study Identification

After conducting an extensive database search, we identified two relevant studies. Despite a thorough examination of references, no additional studies were found. One study was excluded from the review as it did not meet the eligibility criteria; it focused on assessing the utility of intraoperative neuronavigation for a bony resection through anterior approaches to the malformed CVJ [28]. The included study, published in 2014, was a Chinese case series involving 23 patients with CCVJAs, all treated with posterior fixation using intraoperative CT and neuronavigation [29]. The authors reported an overall accuracy rate of 98.1%, with only two misplaced screws, and a 100% arthrodesis rate, without any neurovascular complications. Notably, ten patients underwent occipitocervical fixation using an occipital plate, either due to C1 hypoplasia or limitations imposed by the C2 bone structure.

### 3.3. Illustrative Cases

Case 1 (Figure 1)

A 59-year-old female with a history of chronic hemicrania and severe neck pain was presented at our institution. A CT scan revealed basilar invagination with a dysmorphic dens, along with atlas assimilation. A dynamic MRI confirmed the diagnosis of basilar invagination and assessed the reducibility of the malformation. During positioning, after a distraction and reduction in the malformation, an intraoperative radiograph showed a 6 mm separation between the C0/C1 and C2 facet joints. Our intervention involved decorticating the C0/C1 and C2 facet joints, followed by placing two bilateral 6 mm cages filled with calcium triphosphate (PEEK interbody device; Cervios, DepuySynthes, Raynham, MA, USA). C0/C1 lateral mass-condyle fixation (3.5 × 30 mm) and C2 translaminar fixation (3.5 × 30 mm) were used as anchorage points. After placing the rods, a synthetic bone allograft was applied over the construct. A follow-up CT scan at 58 months showed a mild loss of correction but confirmed the successful arthrodesis at the affected segment.

Case 2 (Figure 2)

A 4-year-old male was presented to our emergency department with progressive tetraparesis. An MRI scan revealed CCVJAs, characterized by basilar invagination along with dens agenesis. T2-weighted sequences showed the spinal cord hyperintensity and a syringomyelic cavity extending from the brainstem to C2. Subsequent CT scans confirmed the MRI findings, highlighting the bifid ventral and dorsal arches of C1, dens agenesis leading to C1–C2 instability, and severe segmental kyphosis. A partial reduction in the dislocation was achieved under fluoroscopic guidance by applying a halo vest, which was maintained for 15 consecutive days with progressively increased traction to enhance reduction. Posterior fixation and arthrodesis were performed with intraoperative neuromonitoring. C1 lateral mass fixation (28 mm length), C2 pedicle fixation (16 mm length), and C3 lateral mass fixation (8 mm length) were established as anchorage points. Rigorous reduction maneuvers, facilitated by rod positioning and securing, resulted in good cervical alignment. A synthetic bone allograft was applied over the surgical site to close the C1 dorsal cleft and promote C1–C3 arthrodesis. A follow-up CT scan at 46 months demonstrated the successful arthrodesis of the C1–C2–C3 segment, closure of the C1 dorsal cleft, and maintenance of the reduction achieved intraoperatively. The patient is now able to walk independently, with a slight tendency toward the internal rotation of the feet and mild motor weakness in the upper limbs.

Case 4 (Figure 3)

A 61-year-old male was presented to our institution with severe and progressively worsening neck pain, along with left-sided brachiocrural hemiparesis that varied with head position. These symptoms developed following two previous head traumas. A CT scan revealed atlas assimilation, basilar invagination, and platybasia. A subsequent dynamic MRI confirmed the basilar invagination, showing a dynamic conflict between the basilar artery/brainstem and the tip of the C2 dens, as well as instability of the left C1–C2 joint. To address these issues, posterior fixation and arthrodesis were performed, using C0/C1 lateral mass-condyle screws (3.5 × 26 mm) and C2 pedicle screws (3.5 × 30 mm) as anchorage points. After placing and securing the rods, an autologous bone graft was harvested from the external diploe of the calvarium. This graft was then secured to the instrumentation using non-resorbable braided sutures, creating an arthrodesis bed between the assimilated C1 arch and the C2 lamina. The patient experienced a complete resolution of symptoms, and a follow-up CT scan at 30 months confirmed the successful CVJ arthrodesis.

Case 8 (Figure 4)

A 45-year-old male with a history of substance abuse (alcohol, cocaine, and psychiatric drugs) and HCV positivity was brought to our emergency department after being struck by a bus. The patient displayed no neurological deficits. A cervical spine CT revealed multiple conditions, including bifid ventral and dorsal arches of C1, Klippel–Feil syndrome at C2–C3 and C5–C6, a Type 3 Anderson–D’Alonzo odontoid fracture at C2, and a left lateral mass fracture at C2. Due to the limited size of the C2 pedicles, C1 lateral mass fixation (3.5 × 34 mm screws) and C3 pedicle fixation (3.5 × 26 mm screws) were selected as anchorage points. A follow-up CT scan at 12 months demonstrated ongoing osteosynthesis, evidenced by the formation of bony trabeculae along the previously fractured lines.

## 4. Discussion

### 4.1. Background

The CVJ is a complex structure involving bones, joints, ligaments, and neurovascular components [30]. Congenital anomalies can alter the biomechanics of this region, leading to instability and biomechanical overload [31,32]. Posterior atlantoaxial fixation (AA) is essential for managing CVJ conditions, and techniques have evolved over time, although concerns such as VA injury remain [33,34]. Goel and Harms [5,35,36,37,38] introduced techniques that allow individual screw placement, reducing anatomical limitations and the risk of VA injury. Various options, including C2 pedicle, pars, and translaminar screws, have been developed to balance screw anchoring with VA safety [17,39].

In cases of hypoplastic C1 or C1 assimilation, surgeons have historically opted for occipitocervical fixation, often using an occipital plate. However, this approach is associated with complications, such as hardware prominence and a high rate of pseudoarthrosis [40], prompting the exploration of alternatives like occipital condyle (C0) fixation [25,41]. Although these approaches offer increased stiffness and a reduced range of motion, they still carry risks, particularly in patients with CCVJAs.

### 4.2. Advancements

The introduction of intraoperative navigation has significantly improved screw insertion success rates while reducing complications. Intraoperative neuronavigation provides a three-dimensional virtual map that merges imaging data with real-time intraoperative visualization. This enhances the surgeon’s ability to plan accurate trajectories, minimize the risk of iatrogenic injury, and improve overall surgical precision [42,43,44].

The surgical series presented here highlights the practical applications and advantages of intraoperative navigation in posterior fixation for CCVJAs. The technology played a crucial role in guiding surgical decisions, enabling precise trajectory planning, and contributing to successful outcomes. It was particularly effective in preventing damage to the VA and hypoglossal nerve during fixation, even in cases where it was preferred over occipital fixation.

The benefits also extended to C2 and C3 pedicle screw fixation, reducing surgical time, blood loss, and complication rates. The outcomes of this study align with those reported by Yu et al., further reinforcing the reliability of intraoperative navigation [29].

### 4.3. Limitations

Despite the advantages, certain limitations must be acknowledged. These include the potential for registration errors, image distortion, and system malfunctions. [45,46,47]. Surgeons must maintain proficiency in interpreting and integrating navigation system data while exercising sound clinical judgment. Accessibility and cost may also present challenges in resource-limited settings, requiring the careful evaluation of feasibility and cost-effectiveness.

The specific limitations of this study include a small patient cohort, the absence of a control group, and a relatively short follow-up duration for some individuals. However, the variability within the pathologies demonstrated the value of navigation in adapting to diverse cases, enabling customized constructs tailored to specific anatomical intricacies.

## 5. Conclusions

In conclusion, our case series and comprehensive literature review highlight the transformative impact of intraoperative navigation on posterior fixation in CCVJAs, representing a significant advancement in surgical precision. Despite challenges related to technical aspects and cost considerations, the advantages of intraoperative navigation are setting a new standard in CCVJA treatment. This technology enables the customization of posterior constructs, a crucial adaptation for addressing anatomical challenges and reducing perioperative neurovascular complications.

In our case series, the effective use of navigation for secure screw placement in the occipital condyles has proven successful in treating CCVJAs, eliminating the need for an occipital plate. Traditionally, occipitocervical constructs that include an occipital plate have been associated with lower fusion rates and increased complication risks. These improvements represent a substantial leap forward, enhancing the safety, precision, and overall outcomes of surgeries in this field, while reducing the need for more invasive and burdensome interventions. As intraoperative navigation becomes increasingly integral, it heralds a new era in the treatment of CCVJAs, offering tailored solutions that address the unique challenges posed by these anomalies.

## Figures and Tables

**Figure 1:**
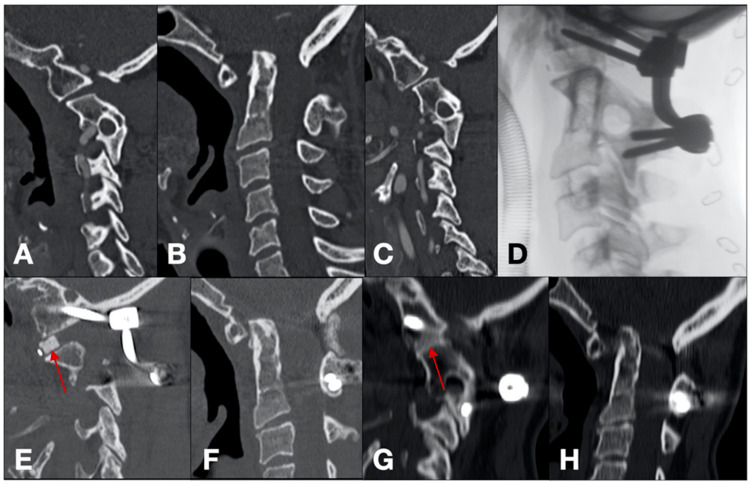
Pre-operative sagittal CT scan (**A**–**C**). Intra-operative radiographic acquisition (**D**). Three-month follow-up CT scan demonstrating correct intra-articular cage positioning (red arrow) and reduction in the malformation (**E**,**F**). 58-month follow-up CT scans demonstrating C0/C1-C2 arthrodesis achievement (red arrow) and mild loss of reduction (**G**,**H**).

**Figure 2:**
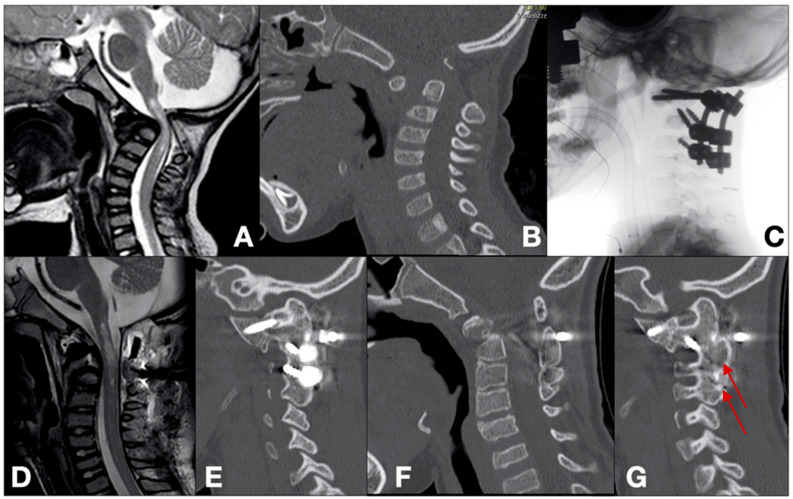
Pre-operative T2-weighted sagittal MRI scan (**A**). Pre-operative sagittal CT scan (**B**). Intra-operative radiographic acquisition (**C**). Post-operative T2-weighted sagittal MRI scan (**D**). 46-month follow-up sagittal CT scan demonstrating correct alignment, reduction in the malformation, and arthrodesis achievement (red arrows) (**E**–**G**).

**Figure 3:**
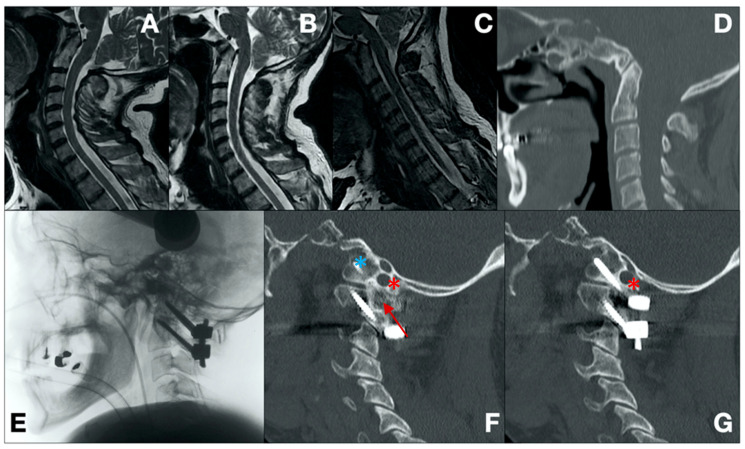
Pre-operative T2-weighted extension, neutral, and flexion sagittal MRI scans, respectively (**A**–**C**). Pre-operative sagittal CT scan (**D**). Intra-operative radiographic acquisition (**E**). 30-month follow-up sagittal CT scans demonstrating correct alignment, arthrodesis achievement (red arrow), and correct screw insertion avoiding critical neurovascular structures (red asterisk for vascular structures and blue asterisk for neural structures) (**F,G**).

**Figure 4:**
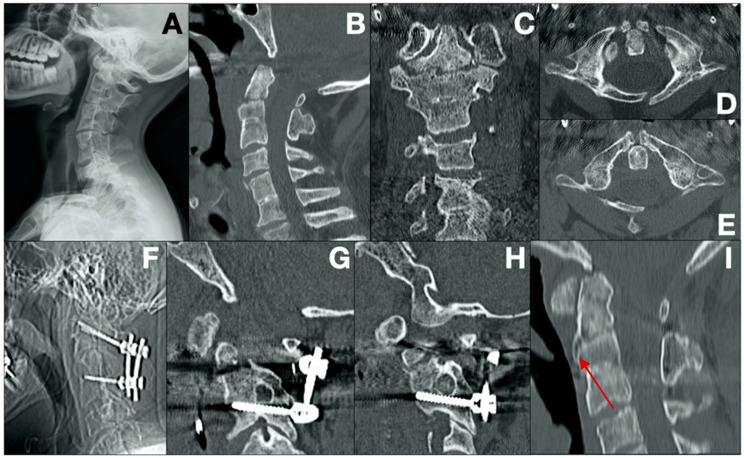
Pre-operative radiographic acquisition (**A**). Pre-operative sagittal, coronal, and axial CT scans, respectively (**B**–**E**). Intra-operative radiographic acquisition (**F**). 12-month follow-up sagittal CT scans demonstrating osteosynthesis achievement (red arrow) and correct screw alignment in the C3 peduncles (**G**–**I**).

**Table 1:** Summary of 8 patients with craniovertebral junction anomalies.

No.	Age(y)/Sex	Main Radiologic Findings	Instrumentation Choice	Complications	Follow-Up (mo)	Fusion Grade
1	59/F	AA, BI	C0/C1–C2 (Laminar) + Bilateral cages	NA	58	1
2	4/M	DA, BA, BI	C1–C2–C3 (articular)	NA	46	1
3	49/M	OO, AAD	C1–C2	NA	35	1
4	61/M	AA, BI, PB	C0/C1–C2	NA	30	1
5	76/M	OO	C1–C2 fixation + subaxial laminoplasty	NA	24	1
6	78/M	AA, AAD	C0/C1–C2–C3 (pedicular)	Dorsal cervicotoracic hematoma	20	1
7	54/M	OO, AAD	C1–C2 + Jazz™ Lock system	NA	16	1
8	54/M	BA, KF, Odontoid Fracture (Type 3 Anderson-D’Alonzo)	C1–C3 (pedicular)	NA	12	1

AA indicates atlas assimilation; BI, basilar invagination; PB, platybasia; OO, os odontoideum; AAD, atlantoaxial dislocation; BA, bifid ventral and dorsal C1 arches; KF, Klippel–Feil syndrome; DA, dens agenesis; NA, not applicable; C0/C1, occipital condyle/lateral mass fixation; C2, axis; C3, third cervical vertebra.

## Data Availability

We excluded the data availability section since our study did not report on any data present in public datasets.

## Abbreviations

The following abbreviations are used in this manuscript:

CCVJA	congenital craniovertebral junction anomalies
CVJ	craniovertebral junction
VA	vertebral artery
